# Prophylactic uterotonics in the prevention of primary postpartum haemorrhage for unplanned out-of-hospital births: a literature review

**DOI:** 10.29045/14784726.2019.03.3.4.15

**Published:** 2019-03-01

**Authors:** Molly Greenaway

**Affiliations:** University of Glasgow

**Keywords:** prophylactic, third stage labour, uterotonic

## Abstract

**Introduction:**

Postpartum haemorrhage (PPH) is a leading cause of maternal morbidity and mortality worldwide. Protocols for the use of prophylactic uterotonics in strategy to prevent PPH have been implemented for in-hospital births following recommendation from the National Institute for Health and Care Excellence (NICE). There are currently no guidelines for prophylactic uterotonic use in out-of-hospital (OOH) births by ambulance crews despite inappropriate birthing conditions and difficulties in obtaining a timely response from community midwives. The aim of this article is to review the use of uterotonic drugs used for the prevention of PPH which could be administered in OOH births.

**Methods:**

The PubMed and ScienceDirect databases were searched for papers discussing the use of prophylactic uterotonics in the third stage of labour, utilising the MeSH keywords: third stage labour, prophylactic, uterotonic. Primary studies, meta-analyses and systematic reviews published between 1998 and 2018 were eligible for inclusion. A review of the full text of the included papers was undertaken using the Critical Appraisal Skills Programme (CASP) checklists.

**Results:**

Of the published articles, 392 were returned, 25 of which met the inclusion criteria. Following assessment of the full text, 11 papers were included for discussion, including a large randomised control trial (WOMAN trial) on the use of tranexamic acid (TXA), which while not a uterotonic drug, was considered a significant drug in the context of PPH management.

**Conclusions:**

PPH is a low incidence, but high risk complication of childbirth. While it is possible for paramedics to administer uterotonics during the third stage of labour, there have been no OOH trials with paramedics to explore whether prophylactic use is safe and effective in the OOH births before arrival (BBA) scenario. Further research is required to determine the efficacy of prophylactic uterotonics in reducing PPH within pre-hospital care.

## Introduction

Unplanned births before arrival (BBA) occurring in the community account for between 0.08% and 1.99% of the overall birth rate within Europe and the United States; a low figure in comparison to in-hospital births (Di Benedetto et al., 1996; McLelland, Morgans, & McKenna, 2014). These cases can be subject to an inappropriate birthing environment, with no immediate option for emergency surgery and the absence of a midwife or obstetrician. Ambulance clinicians are responsible for the management of these births in up to 65% of unplanned out-of-hospital (OOH) births worldwide (McLelland et al., 2014). While basic obstetric training is received, exposure to BBAs is likely to be minimal, since they account for only 0.5% of the total calls per annum (Flanagan, Lord, & Barnes, 2017).

Low clinical exposure to OOH BBA and the unpredictability of intra- and postpartum complications within the community contribute to poorer maternal and newborn outcomes (Bassingthwaighte & Ballot, 2013; Bateman, O’Bryan, Nicholas, & Heagarty, 1994; Bhoopalam & Watkinson, 1991). Although complication rates are low, literature suggests that unplanned BBA are likely to have a statistically significant higher risk of complications and increased perinatal mortality compared to in-hospital deliveries (McLelland, McKenna, Morgans, & Smith, 2018; Snowden et al., 2015).

Paramedics are responsible for managing any complications during this time including cord prolapse, haemorrhage or difficult deliveries. Postpartum haemorrhage (PPH) remains the most common complication observed in OOH BBA and is one of the leading causes of maternal morbidity and mortality worldwide (Hadar et al., 2005; Knight et al., 2016; McLelland et al., 2014), complicating 1.2% of deliveries (Sheldon et al., 2014).

The aim was to review paramedic administration of prophylactic uterotonics during the third stage of labour, and the effect this may have in reducing the incidence and associated morbidity and mortality of PPH.

## Methods

A scoping literature review was performed to explore the use of prophylactic uterotonics in the third stage of labour using two scientific electronic databases: PubMed and ScienceDirect. The search strategy was the use of MeSH keywords: *‘third stage labour’*, *‘prophylactic’*, *‘uterotonic’*. Paper titles were scanned and relevant abstracts were considered for inclusion if they were primary studies, meta-analyses and systematic reviews published between 1998 and 2018 that investigated the prophylactic use of uterotonics (oxytocin, syntometrine and misoprostol) for PPH, administered via all drug routes available to paramedics.

Papers were excluded if they were non-English or reported on healthcare systems in developing countries. Literature that was not subjected to peer-review was discounted. Studies that investigated intra-umbilical administration were removed as this is not, and is unlikely to be, part of paramedic standards of practice.

The author was responsible for scanning and reading articles. If there was an uncertainty as to whether an article should be included, an advisor independent to the review was consulted.

## Results

**Figure 1:**
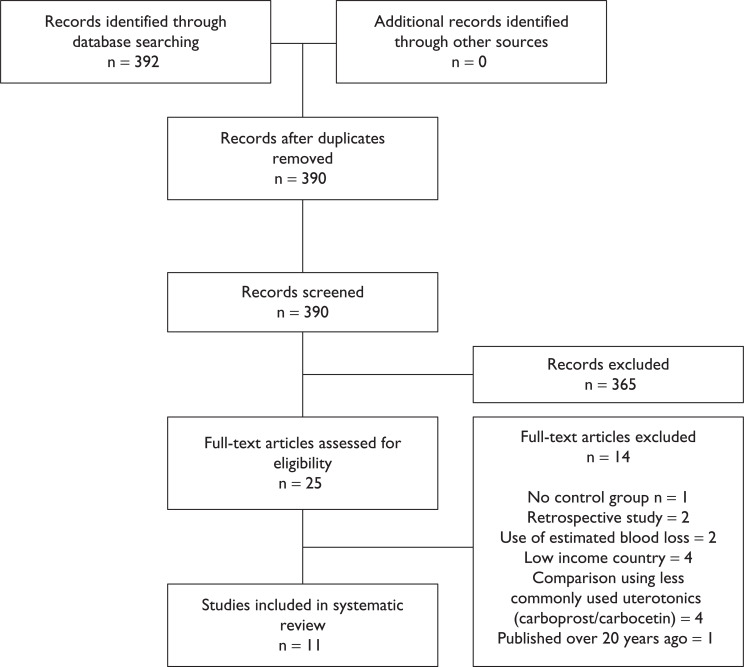
Figure 1. PRISMA diagram of literature search.

The literature search returned 392 results with 25 papers meeting the inclusion criteria (Figure 1). A review of the full text of the included papers was undertaken using the Critical Appraisal Skills Programme (CASP) checklists to assist in identifying the most appropriate papers to contribute to a high quality review (CASP, 2018). This ensured that the papers had clearly addressed a focused question and, in the case of randomised controlled trials, that participants had been selected appropriately. While not part of the original inclusion criteria, the WOMAN trial and a subsequent review and meta-analysis on the use of tranexamic acid (TXA) were also included given its new role in the management of PPH. Following appraisal, 11 papers were considered suitable for inclusion in the review (Table 1).

**Table 1.** Summary of included studies.

Author, year	Aim	Type of study	Intervention	Outcome
Shakur et al. (2017)	Assess the effects of early administration of TXA on death, hysterectomy and other relevant outcomes in women with PPH.	Double-blind, randomised controlled trial.	Tranexamic acid vs. placebo.	Reduced death resulting from PPH, TXA 1.5% vs. placebo 1.9%, p = 0.045.
Prendiville, Harding, Elbourne, & Stirrat (1988)	Compare effect on fetal and maternal morbidity of routine active management of third stage of labour and expectant management.	Randomised controlled trial.	Active vs. expectant management.	Incidence of PPH, active management 5.9% vs. 17.9% in physiological management.
Prendiville, Elbourne, & McDonald (2000)	Assess the effects of active vs. expectant management on blood loss, PPH and other maternal and perinatal complications of the third stage of labour.	Systematic review and meta-analysis.	Randomised trials comparing active vs. expectant management.	Compared to expectant management, active management – PPH > 500 ml – RR 0.38, 95% CI 0.32 to 0.46, maternal blood loss (weighted mean difference −79.33 ml, 95% CI −94.29 to −64.37).
Nørdstrom, Fogelstam, Fridman, Larson, & Rydhstroem (1997)	Compare IV oxytocin administration with saline solution in the management of PPH in the third stage of labour.	Double-blind, randomised controlled trial.	Oxytocin (Partocon 10 IU) vs. 0.9% saline solution.	Oxytocin associated with significant reduction in mean total blood loss (407 vs. 527 ml), frequencies of PPH > 800 ml (8.8% vs. 5.2%), additional treatment with metylergometrine (7.8% vs. 13.8%), postpartum Hb < 10 g/dL (9.7% vs. 15.2%), non-significant increase in frequency of manual placenta removal (3.5% vs. 2.3%).
Sheehan et al. (2011)	Determine effects of adding an oxytocin infusion to bolus oxytocin on blood loss at elective caesarean section.	Double-blind, randomised controlled trial.	Intravenous slow 5 IU oxytocin bolus over 1 minute + additional 40 IU oxytocin infusion in 500 ml of 0.9% saline over 4 hours vs. 5 IU oxytocin bolus over 1 minute + 500 ml of 0.9% saline over 4 hours.	Lower need for additional uterotonic agent in intervention group (12.2% vs. 18.4%), no difference in occurrence of major obstetric haemorrhage between groups (bolus + infusion 15.7% vs. bolus only 16.0%, adjusted OR 0.98, 95% CI 0.77 to 1.25, p = 0.86).
McDonald, Abbott, & Higgins (2004)	Compare the effects of ergometrine-oxytocin with oxytocin in reducing the risk of PPH (blood loss of > 500 ml) and other maternal and neonatal outcomes.	Systematic review and meta-analysis.	Randomised trials comparing ergometrine-oxytocin vs. oxytocin use in third stage of labour managed actively.	Compared with oxytocin, ergometrine-oxytocin was associated with a reduction in risk of PPH (OR 0.82, 95% CI 0.71 to 0.95).
Cotter, Ness, & Tolosa (2001)	Compare effects of oxytocin given prophylactically in third stage of labour on maternal and neonatal outcomes.	Systematic review and meta-analysis.	Randomised or quasi-randomised controlled trials including women anticipating vaginal delivery where oxytocin was given prophylactically for the third stage of labour.	In 7 trials, prophylactic oxytocin reduced blood loss > 500 ml (RR 0.50, 95% CI 0.43 to 0.59) and need for therapeutic oxytocics (RR 0.50, 95% CI 0.39 to 0.64). In 6 trials, little evidence of differential effects for oxytocin vs. ergot alkaloids except oxytocin associated with fewer manual removals of placenta (RR 0.57, 95% CI 0.41 to 0.79). In 5 trials, little evidence of synergistic effect of adding oxytocin to ergometrine vs. ergometrine.
Yuen, Chan, Yim, & Chang (1995)	Compare effect of IM syntometrine and syntocinon in the management of third stage of labour.	Double-blind, randomised prospective trial.	IM syntometrine vs. IM syntocinon.	Syntometrine reduced blood loss postpartum and risk of PPH (OR 0.60, 95% CI 0.21 to 0.88) and need for repeat oxytocin injections (OR 0.63, 95% CI 0.44 to 0.89). Higher incidence of manual removal of placenta when using syntometrine (OR 3.7, 95% CI 1.03 to 1.23).
Gülmezoglu et al. (2001)	Determine whether oral misoprostol is as effective as oxytocin during third stage of labour.	Double-blind, randomised controlled trial.	600 microgram misoprostol orally vs. 10 IU oxytocin IV or IM, in vaginal deliveries.	PPH > 1000 ml greater when given misoprostol (4% vs. 3%, RR 1.39, 95% CI 1.19 to 1.63, p < 0.0001), greater need for additional uterotonics (15% vs. 11%, RR 1.40, 95% CI 1.29 to 1.51, p < 0.0001).
Ng et al. (2001)	Compare effect of oral misoprostol and IM syntometrine in the management of the third stage of labour.	Randomised controlled trial.	600 microgram misoprostol vs. 1 ml syntometrine (500 microgram ergometrine + 5 IU oxytocin).	No significant difference in mean blood loss, incidence of PPH or the fall in Hb concentration. The need for additional oxytocic agents higher in misoprostol group (RR 1.62, 95% CI 1.34–1.96), although manual removal of placenta was reduced (RR 0.29, 95% CI 0.09–0.87).
Li, Gong, Dong, Xie, & Dai (2017)	Assess the efficacy and safety of tranexamic acid in reducing blood loss and lowering transfusion needs for patients undergoing caesarean section or vaginal delivery.	Systematic review and meta-analysis.	Randomised trials comparing IV use of TXA in the intervention group and normal saline or 5% glucose in the control group, in all participants with a singleton pregnancy who underwent an elective caesarean section or intended to deliver vaginally.	TXA results in lower total blood loss in caesarean (154.25 ml, 95% CI −182.04 to −126.47, p < 0.00001) and vaginal deliveries (84.79 ml, 95% CI –109.93 to −59.65, p < 0.00001).

## Discussion

In the UK, guidance on PPH management by paramedics is provided by the Joint Royal Colleges Ambulance Liaison Committee (JRCALC) clinical practice guidelines (JRCALC, 2017). The guidelines advocate administration of syntometrine (or misoprostol if syntometrine is contra-indicated) and TXA when severe haemorrhage occurs following the birth of the baby.

In contrast to in-hospital guidelines which advocate prophylactic administration of uterotonic drugs as part of the active management of the third stage of labour, UK clinical practice guidelines only allow for administration of uterotonics in the management of PPH. Due to the associated risks with OOH PPH and absence of patient safety-netting, the use of pre-hospital prophylactic uterotonics in BBA mothers has been recommended (McLelland et al., 2014).

The components of active management of the third stage of labour are: routine use of uterotonic drugs, delayed cord clamping and controlled cord traction after signs of separation of the placenta. It is recommended to all women by the National Institute for Health and Care Excellence (NICE) due to the reduction in risk of PPH, but is associated with an increased incidence of nausea and vomiting, from 5% with physiological management to 10% with active management (NICE, 2017). However, despite guidelines provided by NICE, 40% of babies still fail to receive delayed cord clamping (after one minute), even in cases where there is no concern about cord integrity and the baby is not showing signs of distress (NICE, 2017; Positive Birth Movement, 2017).

There is some debate about whether mixed management (a combination of both active and physiological management) may be of greater benefit in reducing postpartum blood loss (Begley, Gyte, Devane, McGuire, & Weeks, 2015). This currently requires further research, but is more appropriate to the application of paramedic practice as it may allow for a combination of prophylactic uterotonics, delayed cord clamping and physiological placental delivery.

The Bristol trial demonstrated a 12% reduction of PPH in comparison to physiological management, where uterotonic drugs are not routinely administered (Prendiville, Harding, Elbourne, & Stirrat, 1988). A subsequent meta-analysis by Prendiville, Elbourne, and McDonald (2000), comparing both active and physiological management of PPH, resulted in the International Confederation of Midwives (ICM) and International Federation of Gynecology and Obstetrics (FIGO) supporting the routine use of uterotonics (ICM & FIGO, 2003).

At present, it is unlikely that the entire package of active management will be introduced into routine paramedic practice, although the use of a prophylactic uterotonic is within the paramedic skillset. This is not unjustified given the lack of obstetric training paramedics receive but does raise questions relating to the initial and ongoing training that is required to achieve and maintain competency in the management of childbirth. The scope of paramedic practice is progressing, with in-hospital interventions being undertaken by paramedics in the OOH setting (Eaton, Mahtani, & Catterall, 2018; Kilic et al., 2018; Lyon et al., 2017). Therefore, advances in pre-hospital obstetric management should not be overlooked and OOH guidelines should endeavour to provide equivalent standards of care to those of in-hospital guidelines where practically possible.

### Oxytocin

NICE recommend oxytocin as the first line uterotonic drug in the management of PPH, administered through an intramuscular (IM) injection when the anterior shoulder of the baby is passing, or immediately following delivery. Literature supports the use of oxytocin as it is shown to reduce incidence of PPH, blood loss and subsequent anaemia (Nørdstrom, Fogelstam, Fridman, Larson, & Rydhstroem, 1997).

Nørdstrom and colleagues demonstrated that total PPH blood loss when given oxytocin was significantly less compared with a saline placebo (408 ml vs. 521 ml CI 95% p < 0.001) (Nørdstrom et al., 1997). However, oxytocin was administered by intravenous (IV) infusion, and extrapolating these findings to IM administration is difficult due to differing pharmacokinetics. Sheehan and colleagues (2011) argue that low-dose IV infusion is more effective than IM administration irrespective of the speed of infusion. However, participants in this study were undergoing caesarean section, a procedure expected to experience higher blood loss compared to vaginal delivery, and therefore interpreting these results in other patient groups is ambiguous, and limits its applicability to paramedic practice.

Oxytocin is less suitable to OOH use due to its short half-life of only 10 minutes (Weeks, 2015), necessitating repeated doses (Cotter, Ness, & Tolosa, 2001; McDonald, Abbott, & Higgins, 2004). Furthermore, manufacturers recommend storing oxytocin at 2–8°C which is challenging for ambulance services, adding to the practicalities and economic costs of using this drug.

### Syntometrine

Syntometrine is currently recommended by the UK practice guidelines for active PPH, once the baby has been delivered or in cases of confirmed miscarriage. Evidence suggests syntometrine reduces the risk of minor PPH (> 500–1000 ml) following vaginal delivery for women who are at low risk of PPH in comparison to oxytocin (OR 0.82, 95% CI 0.71 to 0.95, respectively) (McDonald et al., 2004). This risk reduction does not appear to translate to major PPH (> 1000 ml), where no significant difference was seen between syntometrine and oxytocin (OR 0.78, 95% CI 0.58 to 1.03) (McDonald et al., 2004). However, McDonald and colleagues (2004) did not include high-risk patients in their study, which limits this research’s generalisability.

It has also been reported that syntometrine is associated with a higher rate of manual removal of the placenta and while this is statistically significant, incidence remains low (Cotter et al., 2001; Yuen, Chan, Yim, & Chang, 1995). In addition, despite the beneficial effects of syntometrine on reducing PPH, and the reduced need for further uterotonics, commonly reported side effects of syntometrine administration include nausea and vomiting (in up to 46%) and hypertension (a contra-indication if pre-existing). However, oxytocin also has adverse effects including hypotension, cardiac arrhythmias and nausea and vomiting (Dyer, Van Dyk, & Dresner, 2010).

While syntometrine could be administered prophylactically in the third stage of labour there is no clear evidence of benefit in published studies in the pre-hospital setting. In addition, concealed pregnancies and women who have had no antenatal care are encountered by paramedics, which increases the risk of inadvertent administration of uterotonic drugs prior to the birth of a concealed twin. However, multiple births only account for 1.57% of the UK birth rate, and although not impossible, concealed pregnancies are very rare (Office for National Statistics, 2017).

### Misoprostol

Misoprostol is a prostaglandin analogue which has a different mechanism of action to syntometrine (which is via a G-protein coupled receptor), making it usually in suitable for use when syntometrine has been ineffective at achieving haemostasis. Misoprostol has been shown to be effective in preventing PPH but is seen as inferior to oxytocin, owing to the slower onset of action to achieve peak plasma levels (Gülmezoglu et al., 2001). However, Ng and colleagues conducted a randomised controlled trial comparing misoprostol and syntometrine and found no significant difference in the incidence of PPH or mean blood loss (Ng et al., 2001).

Unlike syntometrine, there is no effect on blood pressure, but it can frequently lead to disturbances in body temperature regulation. This can result in body temperatures rising to 40°C, but studies have demonstrated this to be benign and self-limiting with the use of paracetamol (Durocher, Bynum, Leon, Barrera, & Winikoff, 2010). A recent meta-analysis demonstrated that the risk of developing severe hyperthermia is linked to the sublingual route and higher dosing of the drug, likely due to its rapid absorption by the sublingual mucosa and bypass of the first-pass effect through the liver (Elati & Weeks, 2012). Because of this, clinicians have queried the current WHO recommended prophylactic dose of 600 micrograms of misoprostol, since lower doses are shown to be equally effective and associated with fewer side effects (Durocher et al., 2010).

Misoprostol is a promising drug for the prevention of PPH worldwide. Its safety profile and ease of administration allow for easy implementation in to all ambulance services. Misoprostol also has the additional benefit of cost-effectiveness and longevity, making it more appropriate for pre-hospital use. Nonetheless, it is currently likely to remain secondary to syntometrine while the optimal prophylactic dose is debated.

### Tranexamic acid

While not a uterotonic drug, TXA has been added as a further line of management for PPH following positive results seen from large randomised controlled trials (Ker et al., 2012; Shakur et al., 2017). The WOMAN trial was a double-blind randomised controlled trial that evaluated the efficacy of TXA in preventing maternal morbidity and mortality (Shakur et al., 2017). It was shown that both of these were significantly reduced by administration of TXA, although improvements were most evident when drug administration occurred at the earliest possible point following diagnosis of PPH; maternal mortality was reduced by 31% if given within three hours of birth (RR 0.69, 95% CI 0.52–0.91, p = 0.008).

The WOMAN trial also demonstrated that the administration of TXA significantly reduced death due to PPH when used in conjunction with a uterotonic; 96% of both the control and TXA groups received these pharmacological interventions (TXA 1.5% vs. placebo 1.9%, p = 0.045) (Shakur et al., 2017). As a result of this trial, JRCALC approved TXA for use in PPH in 2017, but it is awaiting implementation by many Trusts.

There remains debate regarding the appropriate dosing, although the WOMAN trial demonstrated a fixed dose of 1g administered at a rate of 100 milligrams per minute of TXA is more appropriate in an emergency setting and excludes the need of accurately knowing patient weight.

Efforts have been made to evaluate the use of TXA as an effective prevention for PPH. However, currently there is insufficient evidence to support its use prophylactically and a randomised controlled trial would be required to draw conclusions on this (Li, Gong, Dong, Xie, & Dai, 2017); its use has therefore been restricted to those who are actively bleeding (JRCALC, 2017).

Although there are other drugs available for in-hospital use (ergometrine, carboprost), it is unlikely that where a protocol does not yet exist for the prophylactic management of PPH in pre-hospital care, implementation is unlikely to involve drugs that are not currently in the paramedic standards of practice.

## Conclusion

PPH is a low incidence, but high risk complication of childbirth. While it is possible for paramedics to administer uterotonics for the management of PPH as recommended by NICE, there have been no OOH trials with paramedics to explore prophylactic administration in the third stage of labour in the OOH BBA scenario. Further research is required to determine the safety and efficacy of prophylactic uterotonics in reducing PPH within pre-hospital care.

## Conflict of interest

None declared.

## Funding

None.
